# The kinase domain residue serine 173 of *Schizosaccharomyces pombe* Chk1 kinase is critical for the response to DNA replication stress

**DOI:** 10.1242/bio.029272

**Published:** 2017-11-01

**Authors:** Naomi Coulton, Thomas Caspari

**Affiliations:** 1 Genome Biology Group, Bangor University, School of Medical Sciences, Bangor LL57 2UW, UK; 2 Postgraduate School, Paracelsus Medical University, Strubergasse 21, 5020 Salzburg, Austria

**Keywords:** Chk1, Kinase, Cell cycle, DNA replication, Checkpoint

## Abstract

While mammalian Chk1 kinase regulates replication origins, safeguards fork integrity and promotes fork progression, yeast Chk1 acts only in G1 and G2. We report here that the mutation of serine 173 (S173A) in the kinase domain of fission yeast Chk1 abolishes the G1-M and S-M checkpoints with little impact on the G2-M arrest. This separation-of-function mutation strongly reduces the Rad3-dependent phosphorylation of Chk1 at serine 345 during logarithmic growth, but not when cells experience exogenous DNA damage. Loss of S173 lowers the restrictive temperature of a catalytic DNA polymerase epsilon mutant (*cdc20.M10*) and is epistatic with a mutation in DNA polymerase delta (*cdc6.23*) when DNA is alkylated by methyl-methanesulfate (MMS). The *chk1-S173A* allele is uniquely sensitive to high MMS concentrations where it displays a partial checkpoint defect. A complete checkpoint defect occurs only when DNA replication forks break in cells without the intra-S phase checkpoint kinase Cds1. Chk1-S173A is also unable to block mitosis when the G1 transcription factor Cdc10 (*cdc10.V50*) is impaired. We conclude that serine 173, which is equivalent to lysine 166 in the activation loop of human Chk1, is only critical in DNA polymerase mutants or when forks collapse in the absence of Cds1.

## Abstract (precis)

**Summary:** Mutation of serine-173 in the kinase domain of Chk1 increases genomic instability as it abolishes the response to DNA lesions that arise while chromosomes are being copied.

## INTRODUCTION

*Schizosaccharomyces* *pombe* Chk1 is phosphorylated in its C-terminal domain at serine-345 by Rad3 (ATR) after the kinase is recruited to a broken chromosome by Rad4 (TopBP1), Crb2 (53BP1) and the Rad9-Rad1-Hus1 ring (Capasso et al., 2002; Lopez-Girona et al., 2001; Saka et al., 1997; Furuya et al., 2004). Activated Chk1 delays cell-cycle progression at the G2-M boundary by stimulating Wee1 to phosphorylate the inhibitory tyrosine-15 residue of Cdc2 (CDK1) kinase and by simultaneously removing the activating tyrosine phosphatase Cdc25 from the nucleus (Furnari et al., 1999; O'Connell et al., 1997). It is generally believed that the Chk1 response in yeast is limited to G2 even though DNA replication forks collapse in S phase (Lindsay et al., 1998; Francesconi et al., 1993; Redon et al., 2003). *S. pombe* Chk1 performs a second, more enigmatic role in G1 where it prevents premature mitosis when the transcription factor Cdc10 is impaired (Carr et al., 1995). It also phosphorylates Cdc10 in the presence of methyl-methanesulfonate (MMS) that alkylates the DNA template to delay G1-S transition (Ivanova et al., 2013).

Unlike in yeast, human Chk1 acts mainly during S phase. It is also phosphorylated at S345 by ATR when the kinase associates with stalled DNA replication forks via Claspin (Mrc1), in a process aided by TopBP1 (Rad4) and the 9-1-1 ring. Modification of S345 depends on the additional phosphorylation of S317 and is followed by the auto-phosphorylation of Chk1 at S296 (reviewed in González Besteiro and Gottifredi, 2015). This auto-phosphorylation event is important for the association of Chk1 with Cdc25A and the subsequent degradation of the phosphatase (Kasahara et al., 2010). Modification of S280 by p90 RSK kinase ensures the nuclear localisation of Chk1 (Li et al., 2012). Human Chk1 associates also with DNA lesions independently of Claspin by binding to poly-ADP-ribosyl modified PARP (Min et al., 2013). Activated Chk1 blocks late replication origins by disrupting the TopBP1-Treslin complex, promotes translesion DNA polymerases, mediates homologous recombination at broken forks through Rad51 and BRCA2, regulates fork elongation and arrests cell cycle progression by promoting the degradation of Cdc25A (reviewed in González Besteiro and Gottifredi, 2015). While yeast Chk1 can be deleted (Walworth and Bernards, 1996), mammalian cells depend on the kinase for viability. Interestingly, only S345 phosphorylation is required for the essential roles of Chk1 (Wilsker et al., 2008). Inhibition of human Chk1 in unperturbed cells interferes with S phase (Petermann et al., 2010) and mitosis (Zachos and Gillespie, 2007). Cdc2 (CDK1) phosphorylates human Chk1 at S286 and S301 during normal mitosis as well as in the response to DNA damage (Shiromizu et al., 2006; Ikegami et al., 2008) with as yet unknown functional implications.

Another open question is how the catalytic activity of Chk1 is regulated. The generally accepted model predicts an auto-inhibitory complex between the N-terminal kinase domain and the C-terminal regulatory domain (Kosoy and O'Connell, 2008; Palermo et al., 2008). This complex is thought to open up when S345 is phosphorylated by ATR (Rad3) at sites of DNA damage. Whether this model is correct is still unclear since only the N-terminal kinase domain of human Chk1 has been crystallised (Chen et al., 2000). The activation loop adopts an open conformation in this structure which implies that Chk1 does not depend on the modification by an upstream activator as many other kinases do. How Chk1 is silenced at the end of the DNA damage response is also not fully understood. Human Chk1 is degraded after its modification at S345 in a process that is independent of the other phosphorylation sites (Zhang et al., 2005). A similar degradation does not occur in yeast. Attenuation of Chk1 correlates with its dephosphorylation at S345 by Wip1 (PPM1D) in human cells (Lu et al., 2005) and by Dis2 in *S. pombe* (den Elzen and O'Connell, 2004). Interestingly, Wip1 is replaced by PPA2 in undamaged cells where it dephosphorylates Chk1 at S317 and S345 (Leung-Pineda et al., 2006). Currently no information is available on the regulation of Chk1 in unperturbed yeast cells.

We report here a rare separation-of-function mutation in Chk1 kinase. Mutation of serine 173 (S173A) in the kinase domain of *S. pombe* Chk1 abolishes the G1-M arrest, when cells arrest at start in a leaky *cdc10.V50* mutant stain, and the S-M arrest when DNA replication forks break in the absence of the intra-S checkpoint kinase Cds1 (Chk2). The G2-M checkpoint responses are largely intact with the exception of high MMS concentrations. Interestingly, the mutant kinase is fully phosphorylated by Rad3 at S345 upon exogenous DNA damage, but only at a very low level when logarithmically growing cells experience endogenous replication stress. The specific MMS sensitivity of *chk1-S173A* cells is epistatic with a mutation in the catalytic domain of the lagging strand DNA polymerase delta. Since S173 is equivalent to lysine 166 in the activation loop of human Chk1, a residue important for substrate recognition (Chen et al., 2000), we conclude that the corresponding section of *S. pombe* Chk1 is critical for the recognition of a DNA replication protein.

## RESULTS

### Reduced S345 phosphorylation of Chk1-S173A in unperturbed cells

**Fig. 1.**
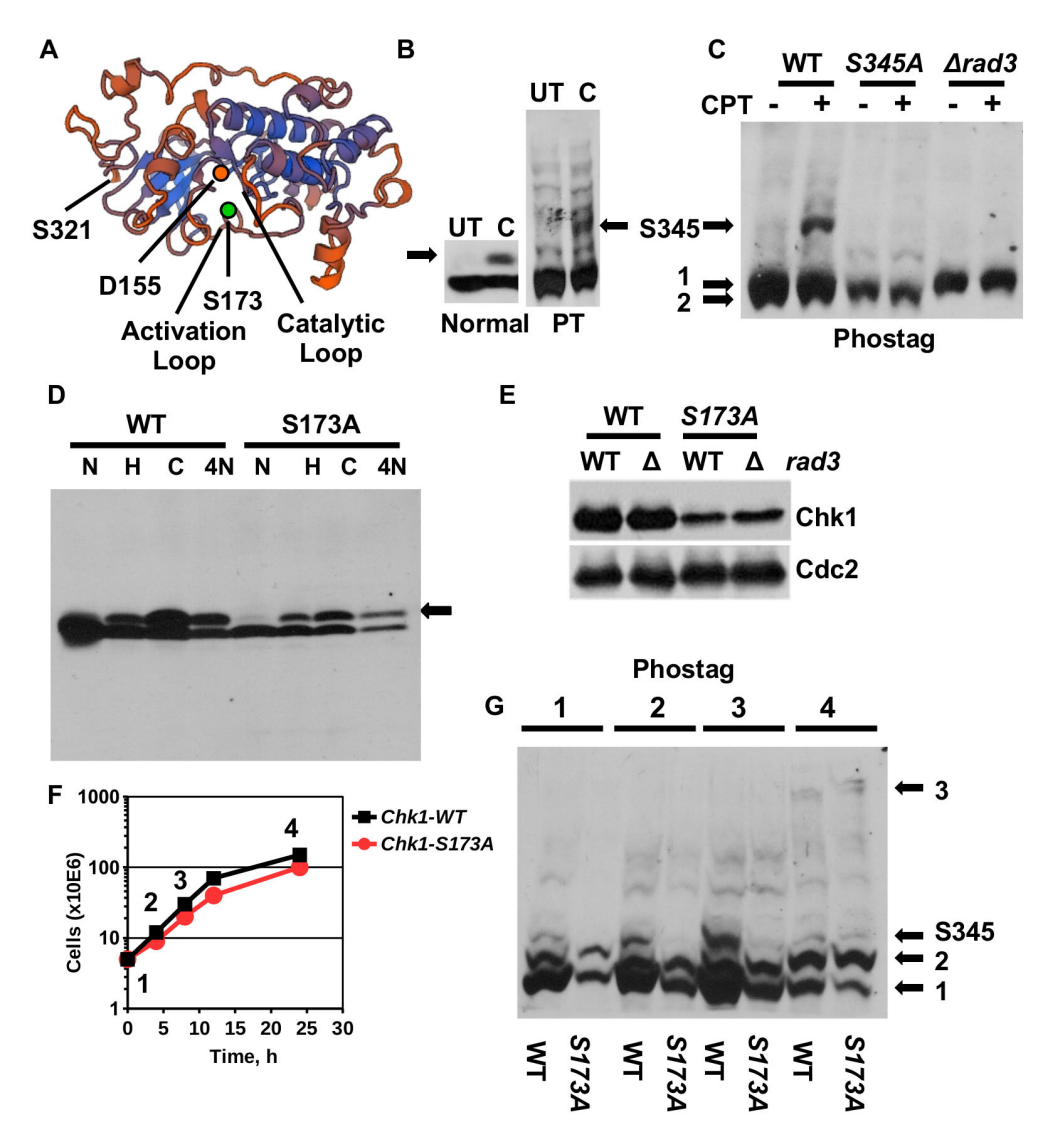
**Reduced S345 phosphorylation of Chk1-S173A in unchallenged cells.** (A) Model of the kinase domain of *S. pombe* Chk1. The Swiss model tool was used (https://swissmodel.expasy.org). The underlying crystal structure is 4czt (34.5% identity) (Chaves-Sanjuan et al., 2014). Serine-321 is the last C-terminal amino acid. (B) *chk1-HA_3_* wild-type cells were treated in rich medium with 10 μM camptothecin (CPT) for 3.5 h at 30°C. UT, untreated. Total protein extracts were separated on normal 10% SDS page or 6% phostag SDS page (PT). The arrow indicates the group of shift bands related to S345 phosphorylation. (C) PT-SDS page showing extracts from *chk1-HA_3_*, *chk1-S345A-HA_3_* and *chk1-HA_3_ rad3::ade6+* cells treated with 10 μM CPT for 3.5 h. Numbers 1 and 2 indicate the hypo-phosphorylated double band. (D) Normal SDS page analysis of *chk1-HA_3_* and *chk1-S173A-HA_3_* cells treated with 12 mM hydroxyurea (HU) and 10μM CPT for 3.5 h or with 10 µM nitroquinoline 1-oxide (4NQO) for 1 h at 30°C (full image shown in Fig. S2B) (Chk1 runs at 58 kDa). (E) PT-SDS page analysing extracts from untreated *chk1-HA_3_*, *chk1-HA_3_ rad3::ade6+*, *chk1-S173A-HA_3_* and *chk1-S173A-HA_3_ rad3::ade6+* cells. (F,G) Untreated *chk1-HA_3_* and *chk1-S173A-HA_3_* cells were grown in rich medium from a low cell number into stationary phase. Samples were withdrawn at the indicated time points and analysed on PT-SDS page. 1 and 2 indicate the Chk1 double band. The two bands labelled with 3 appear specifically in non-growing cells.

Lysine 166 occupies a central position in the activation loop of human Chk1 opposite the catalytic aspartate 130 (D155 in *S. pombe*, Fig. 1A) where it may determine substrate specificity (Chen et al., 2000). The corresponding *S. pombe* residue is serine 173 (Fig. 1A) and aspartate 189 in *Saccharomyces* *cerevisiae*.

To find out whether S173 plays a role in Chk1 activity, we mutated this residue to alanine and integrated the mutant gene with a C-terminal HA_3_ tag (*chk1-S173A-HA_3_*) at its endogenous locus using the Cre-*lox* recombination system (Watson et al., 2008). The integrated gene was amplified and the mutation was confirmed by DNA sequencing. We also integrated the wild-type gene (*chk1-HA*_3_) (Walworth and Bernards, 1996) to exclude any effects of the flanking *lox* DNA sequences on *chk1* expression (Fig. S1).

We first used the phos-tag electrophoresis assay (Caspari and Hilditch, 2015) to study the phosphorylation pattern of wild type Chk1 to establish a base line for the analysis of Chk1-S173A. Phos-tag acrylamide slows down the mobility of proteins relative to the extent of their phosphorylation (Kinoshita et al., 2006). We activated wild-type Chk1 with the topoisomerase 1 inhibitor camptothecin (CPT) that breaks DNA replication forks in S phase (Pommier et al., 2010). As previously reported (Wan et al., 1999), CPT induced the mobility shift of Chk1-HA on normal SDS page which is triggered by the phosphorylation of S345 by Rad3 (ATR) (Capasso et al., 2002) (Fig. 1B). Analysis of the same samples on a phos-tag gel revealed a larger number of phosphorylated Chk1 forms in untreated cells and a group of additional bands when cells were treated with 10 μM CPT for 3.5 h (Fig. 1B,C). Since these inducible bands were absent in the S345A mutant (*chk1-S345A-HA_3_*) (Janes et al., 2012) and in cells without Rad3 kinase (*chk1-HA_3_ Δrad3*), they are related to the phosphorylation of serine 345 (Fig. 1C). We also noticed that the hypo-phosphorylated material of Chk1 at the bottom of the phos-tag gel consists of at least two bands (1 and 2 in Fig. 1C). Intriguingly, deletion of Rad3 kinase and mutation of its phosphorylation site S345 affected the two bands 1 and 2 differentially. While loss of the phosphorylation site advanced the mobility (i.e. reduced phosphorylation), inactivation of Rad3 had the opposite effect (i.e. increased phosphorylation) (Fig. 1C; Fig. S2A). This implies that other kinases gain access to Chk1 in the absence of Rad3. Mutation of S173 to alanine (S173A) had no obvious impact on the normal band shift when cells were treated with 12 mM hydroxyurea (HU), which stalls DNA replication forks, with 10μM CPT or with the UV mimetic 4-nitroquinoline 1-oxide (4-NQO) at 10 μM (Fig. 1D). It resulted, however, in a decrease in the total amount of Chk1 (Fig. 1E; Fig. S2B).

**Fig. 2.**
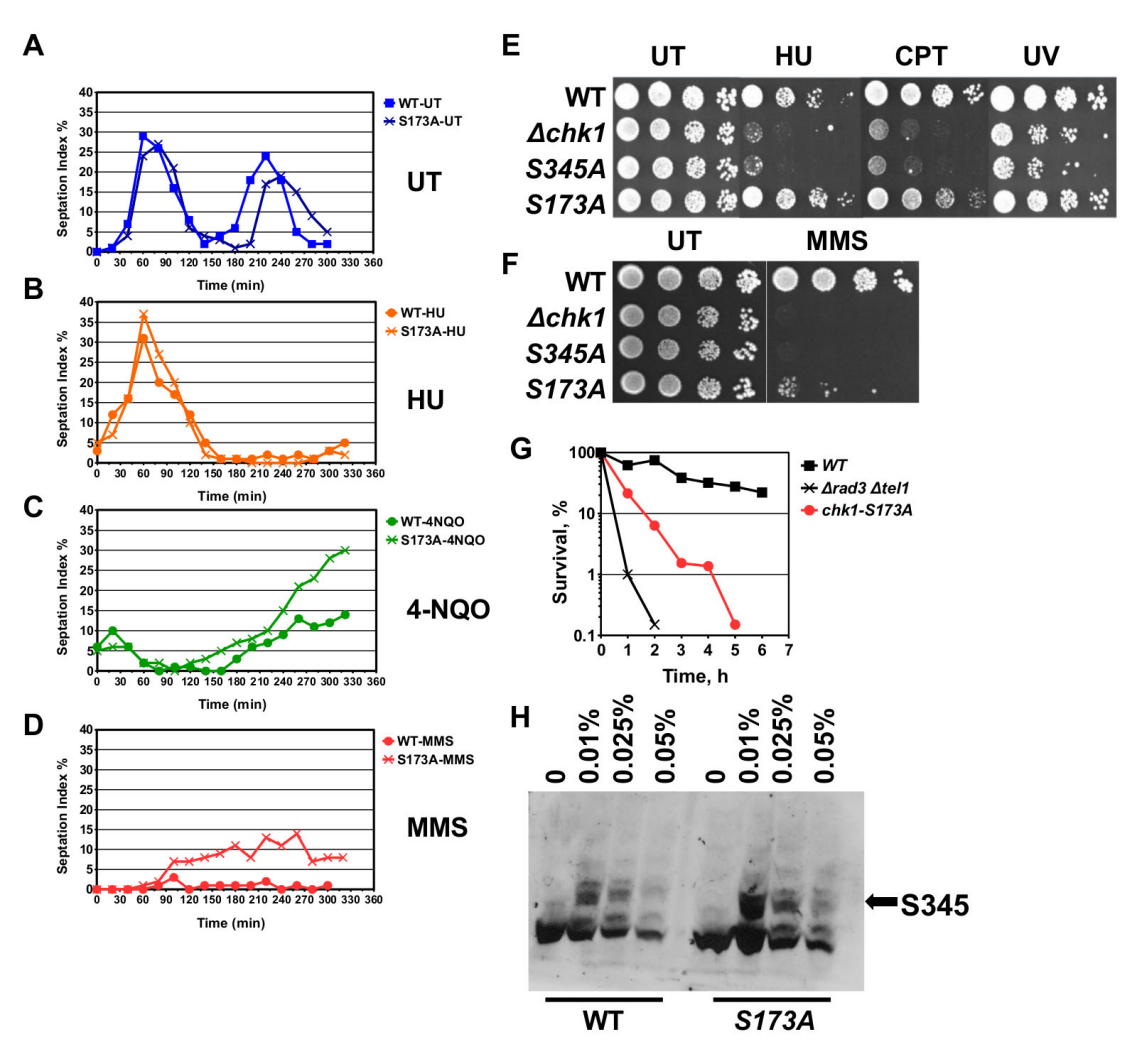
**Chk1-S173A cells are MMS sensitive.** (A-D) *chk1-HA_3_* and *chk1-S173A-HA_3_* cells were synchronised in G2 by lactose gradient centrifugation and released into rich medium containing no drug (UT), 12 mM hydroxyurea (HU), 10 µM nitroquinoline 1-oxide (4NQO) or 0.05% methyl-methanesulfonate (MMS). (E,F) Drop test of the indicated strains on rich medium plates containing 4 mM HU, 10 μM CPT, 0.01% MMS or were treated with 50 J/m^2^ UV light (254 nm). (G) Acute cell survival at 0.05% MMS. The *rad3::ade6+ tel1::leu2+* double mutant is checkpoint defective (averages of 3 repeats). (H) PT-SDS page analysis of total protein extracts from *chk1-HA_3_* and *chk1-S173A-HA_3_* cells treated with the indicated MMS concentrations for 3.5 h at 30°C in rich medium.

To find out whether the unperturbed phosphorylation of Chk1 relates to cell physiology, we grew cells from an early logarithmic state into stationary phase and withdrew samples at different times (Fig. 1F). The band associated with S345 phosphorylation peaked during the most active growth phase of wild-type cells (time points 2 and 3 in Fig. 1G) and was absent once cells had exited the cell cycle (time point 4 in Fig. 1G). This endogenous S345 phosphorylation of Chk1 reflects most likely the occurrence of DNA replication damage. It was, however, very interesting to find that the S173A mutation almost abolished S345 phosphorylation during the unperturbed growth phase (Fig. 1G; Fig. S2C,D). Given that S345 is fully phosphorylated when *chk1-S173A* cells experience exogenous DNA damage (Figs 1D and 2H), we concluded that S173 is critical for checkpoint signals during an unchallenged S phase but not when exogenous DNA lesions occur. It was also interesting to find that highly phosphorylated forms (double band 3 in Fig. 1G) accumulated in stationary phase.

### Chk1-S173A cells are sensitive to DNA alkylation

Since Chk1 is crucial for the G2-M checkpoint (Walworth and Bernards, 1996), we synchronised *chk1-HA_3_* wild-type and *chk1-S173A-HA_3_* cells in G2 by lactose gradient centrifugation (Luche and Forsburg, 2009) and released them into rich medium with or without MMS (0.05%), 4NQO (10 μM) or HU (12 mM) at 30°C to measure the delay time. The first telling observation came when we compared the untreated strains. While wild-type cells (*chk1-HA_3_*) entered the second cycle at around 180 min, *chk1-S173A-HA_3_* cells were delayed by 20 min (Fig. 2A). Such a second cycle delay is typical for agents like CPT or HU which interfere with DNA replication (Mahyous Saeyd et al., 2014). It is therefore possible that the *chk1-S173A-HA_3_* strain suffers from a DNA replication problem that triggers this short G2 delay. The UV mimetic 4-NQO and the DNA alkylation agent MMS blocked both the passage through the first G2 since DNA is instantly damaged, whereas HU caused the expected second cycle arrest as cells are only hit once they undergo DNA replication (Lindsay et al., 1998). While the S173A mutation had no impact on the HU arrest (Fig. 2B), it allowed cells to exit G2 slightly earlier in the presence of 4-NQO and MMS (Fig. 2C,D). This small G2-M checkpoint defect was more prominent for the high MMS concentration as *chk1-S173A-HA_3_* cells started to return to the cell cycle already after 80 min compared with wild-type cells which arrested throughout the experiment (Fig. 2D). This checkpoint defect correlated with a MMS sensitivity (0.05%) of the mutant strain (Fig. 2F,G). Interestingly, a similar loss of viability was not observed when the *chk1-S173A-HA_3_* strain was treated with HU, CPT or UV light (Fig. 2E). This is an important finding as it reveals S173A as a separation-of-function mutation. MMS modifies both guanine (to 7-methylguanine) and adenine (to 3-methlyladenine) thereby inducing mismatches in the DNA that are repaired by base excision repair. Ineffective base excision repair (BER) results in single-stranded DNA breaks independently of the cell cycle but causes DNA double-strand breaks when these gaps are encountered by a replication fork (Lundin et al., 2005). The MMS sensitivity of the *chk1-S173A-HA_3_* mutant was not related to a defect in S345 phosphorylation as the mutant kinase displayed the characteristic band shift on phos-tag SDS page in a concentration rage from 0.01% to 0.05% MMS (Fig. 2H). Interestingly, the S345 shift was strongest at the lowest MMS concentration of 0.01% and declined at the higher concentrations.

### The MMS defect of Chk1-S173A is dose-dependent

**Fig. 3.**
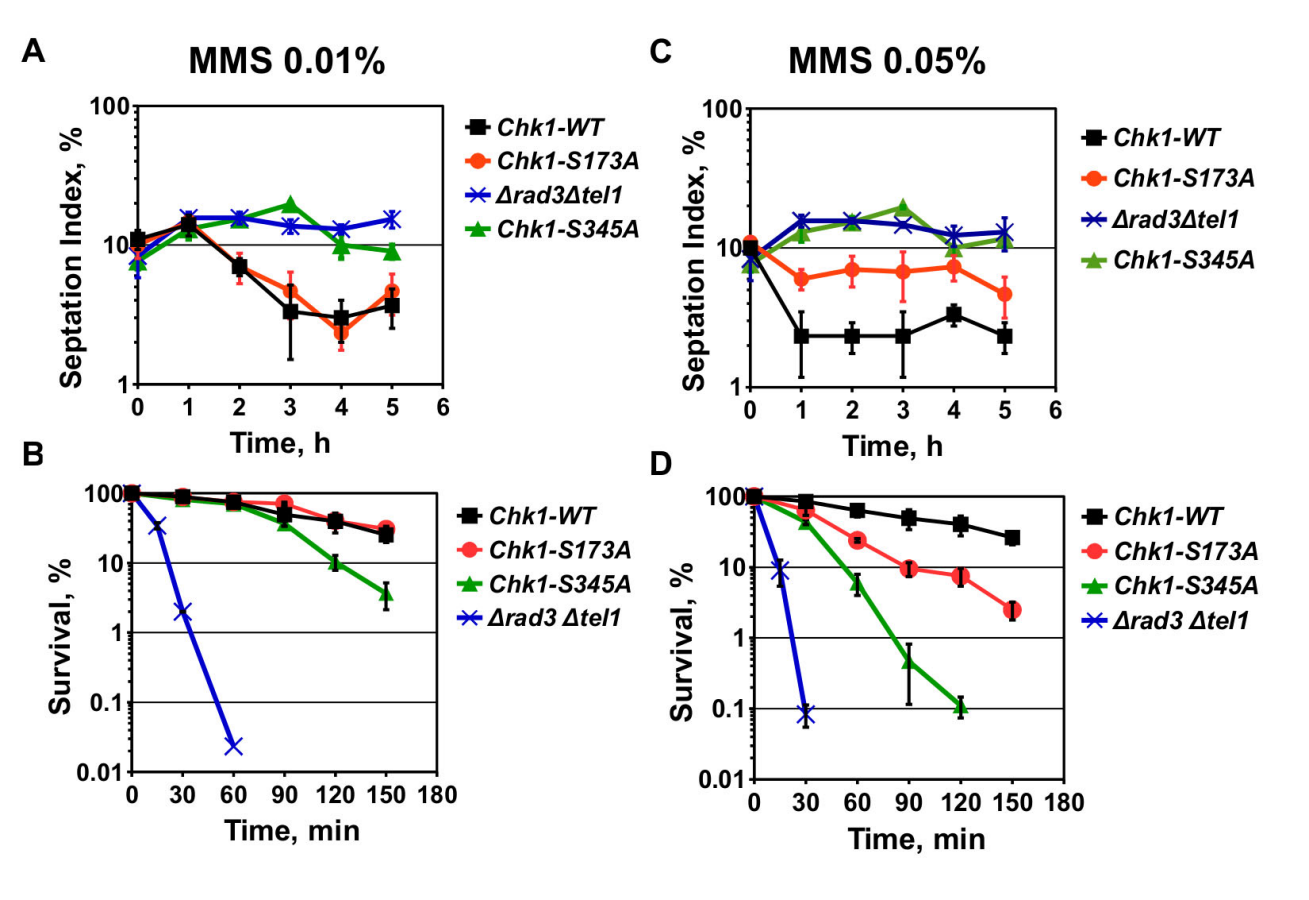
**The MMS defect of Chk1-S173A is dose-dependent.** (A,C) The indicated strains were treated with 0.01% or 0.05% MMS over a period of 5 h. Samples were withdrawn, fixed in methanol and septated G1/S cells were scored. (B,D) Survival of the same strains was analysed over a period of 2.5 h. Mean±s.d. of three repeats are shown throughout.

Since phosphorylation of S345 was stronger at the lower MMS concentration of 0.01% compared to 0.05% at which the *chk1-S173A* mutant is sensitive and partly checkpoint defective (Fig. 2H), we analysed cell cycle progression and sensitivity again at 0.01% and 0.05%. To avoid any interference by the synchronisation protocol, we added MMS directly to asynchronous cultures and scored the number of septated G1/S cells. As previously reported (Lopez-Girona et al., 2001), *chk1-S345A* and checkpoint defective *Δrad3Δtel1* strains failed to arrest at both MMS concentrations as their septation index remained high (Fig. 3A,C). While the *chk1-S173A* strain was partly checkpoint defective at 0.05% MMS, consistent with the previous experiment (Fig. 2D), this defect was absent at the lower concentration of 0.01%. This concentration dependency was reflected in the MMS sensitivity. In contrast to the *chk1-S345A* and *Δrad3Δtel1* strains, *chk1-S173A* cells only lost viability at 0.05% (Fig. 3B,D). Since MMS produces single-strand lesions which inhibit DNA replication and induces gene expression in a dose-dependent manner (Benton et al., 2006), the S173A mutation becomes problematic only when too many lesions affect the cell.

### Chk1-S173A is defective in the G1-M checkpoint

**Fig. 4.**
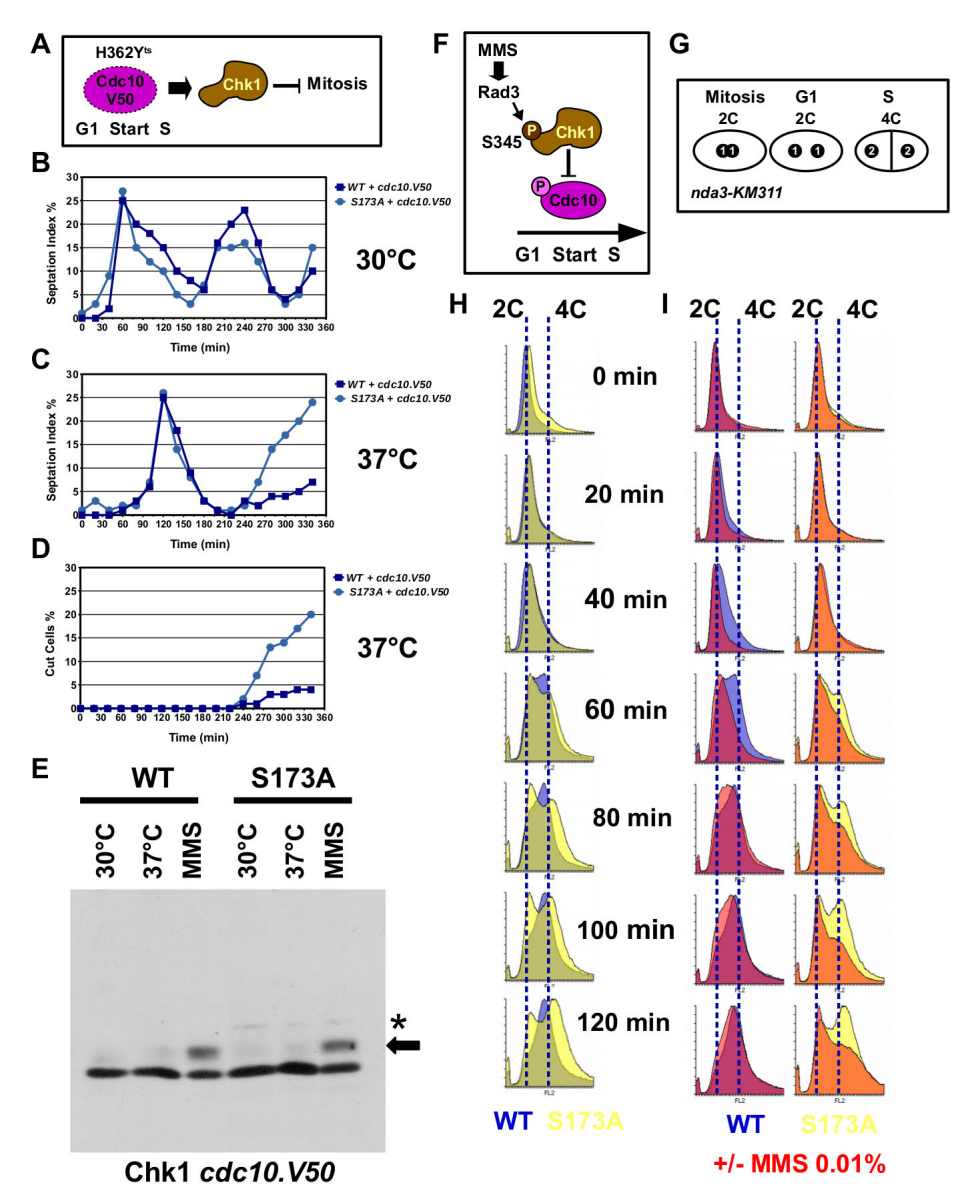
**Chk1-S173A is defective in the G1-M checkpoint.** (A) Inactivation of Cdc10 induces a Chk1-dependent block of mitosis. (B-D) *chk1-HA_3_ cdc10.V50* and *chk1-S173A-HA_3_ cdc10.V50* cells were grown in rich medium at 30°C, synchronised in G2 by lactose gradient centrifugation and released into rich medium at 30°C (B) or 37°C (C). Note the 60 min delay of the first cycle due to the temperature up-shift. The majority of *chk1-S173A-HA_3_ cdc10.V50* cells enter a terminal mitosis (cut phenotype) (D). (E) *chk1-HA_3_ cdc10.V50* and *chk1-S173A-HA_3_ cdc10.V50* cells were grown in rich medium at 30°C, at 37°C or at 30°C with 0.01% MMS for 3.5 h. Total protein extracts were analysed on normal SDS page. (F) MMS treatment delays G1-S transition by activating Rad3-Chk1 phosphorylation of Cdc10. (G) The cold-sensitive beta-tubulin gene *nda3.KM311* arrests cells with a 2×1C=2C DNA content in metaphase. Cells reach a 4C DNA content after S phase. (H) Flow cytometry histograms of untreated *chk1-HA_3_ nda3.KM311* (blue) and *chk1-S173A-HA_3_ nda3.KM311* cells (yellow) after release from the metaphase block in rich medium. Time is post-release. (I) Flow cytometry histograms of MMS-treated (0.01%) cells after the release from the metaphase block. Dark colours show plus 0.01% MMS. Blue and yellow show untreated. The 2C and 4C DNA content is indicated by dotted lines.

In addition to its key role in G2, Chk1 blocks mitosis when *S. pombe* cells arrest at start in a *cdc10.V50* mutant (Fig. 4A) (Carr et al., 1995). Cdc10 is a subunit of the MBF transcription factor complex that activates S-phase genes during the G1-S transition (Lowndes et al., 1992). We constructed *chk1-HA_3_* and *chk1-S173A-HA_3_* double mutants with the temperature-sensitive *cdc10.V50* (H362Y) allele (Marks et al., 1992) and released G2-synchronised cells into rich medium at 30°C and 37°C (Fig. 4B,C). As reported previously (Carr et al., 1995), *chk1-HA_3_ cdc10.V50* cells progressed through the first cycle before arresting in G2 at the restrictive temperature of 37°C (Fig. 4C). Entry into the first cycle was delayed by 60 min due to the increase in the temperature (Janes et al., 2012). While *chk1-HA_3_ cdc10.V50* cells leaked slowly out of this G2-M arrest with only a few cells displaying the terminal cut phenotype where the new cell wall cuts through the nucleus, *chk1-S173A-HA_3_ cdc10.V50* cells entered mitosis much faster with most cells showing the cut phenotype (Fig. 4C,D). We concluded from this experiment that the mutation of S173 impairs the G1-M checkpoint function of Chk1. Interestingly, this G1-M function of Chk1 is independent of its S345 phosphorylation as the temperature up-shift from 30°C to 37°C did not trigger the band shift on normal SDS page (Fig. 4E). We decided to utilise the leaky *cdc10.V50* allele, where cells slowly exit the G1 arrest at 37°C to enter mitosis in a Chk1-dependent manner, instead of the stronger *cdc10.M17* allele (Willis and Rhind, 2011) as the same allele was used in the earlier study by Carr et al. (1995).

Since Chk1 acts also upstream of Cdc10 to prevent entry into S phase when the DNA template is alkylated by MMS (Fig. 4F) (Ivanova et al., 2013), we synchronised *chk1-HA_3_* and *chk1-S173A-HA_3_* cells in metaphase using the cold-sensitive *nda3.KM311* allele (Hiraoka et al., 1984) and released cells into rich medium with or without 0.01% MMS by raising the temperature from 20°C to 30°C. This experiment would allow us to measure the delay in G1-S transition induced by MMS. Untreated wild-type cells (*chk1-HA_3_ nda3.KM311*) initiated DNA replication between 40 min and 60 min post-release which increased the DNA content from 2C to 4C (Fig. 4G,H). The mutant strain (*chk1-S173A-HA_3_ nda3.KM311*) showed a similar behaviour, but displayed one interesting difference: not all cells were able to escape the mitotic arrest as they maintained a 2C DNA content (Fig. 4H). The delayed exit from the metaphase arrest could be linked with the ability of *S. pombe* Chk1 to sustain the activation of the spindle checkpoint that delays metaphase-to-anaphase transition (Collura et al., 2005). Hence, the S173A mutation may prolong this mitotic arrest. The addition of MMS delayed the accumulation of the 4C DNA content in both strains, with the S173A mutant showing a more pronounced effect (Fig. 4I). This led us to conclude that the S173A mutation affects only the down-stream function of Chk1 that restrains mitosis in the *cdc10.V50* mutant, but not the up-stream function which delays G1-S transition in the presence of MMS.

### Chk1-S173A fails to respond to broken replication forks in the absence of Cds1

**Fig. 5.**
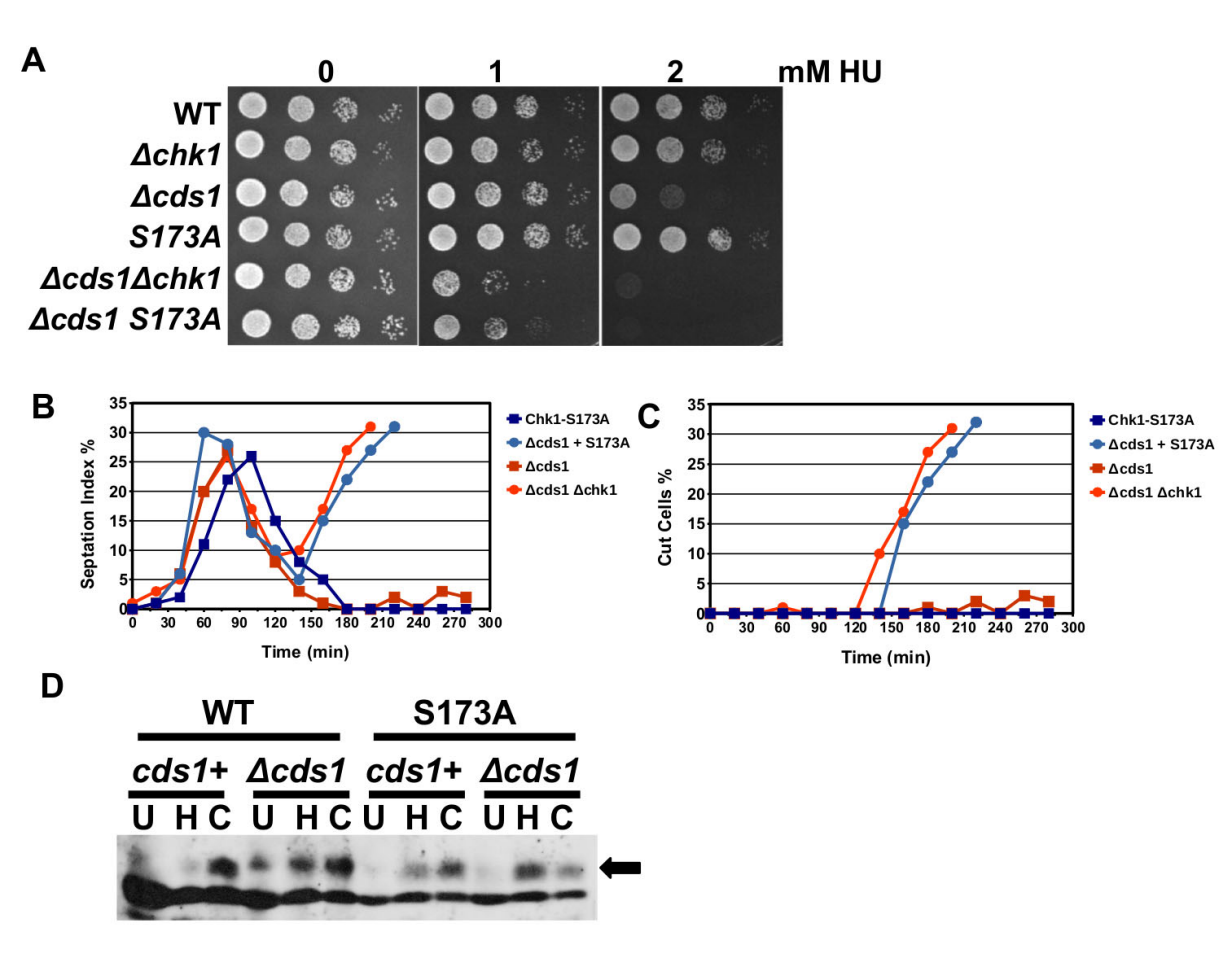
**Chk1-S173A fails to respond to broken replication forks.** (A) Drop test with the indicated strains at 30°C on rich medium plates. (B,C) *chk1-S173A-HA_3_*, *chk1-S173A-HA_3_ cds1::ura4+*, *cds1::ura4+* and *cds1::ura4+ chk1::kanMX4* strains were synchronised by lactose gradient centrifugation and released into rich medium with 12 mM HU at 30°C (B, sepated G1/S cells; C, cut cells). (D) *chk1-HA_3_*, *chk1-HA_3_ cds1::ura4+*, *chk1-S173A-HA_3_*, *chk1-S173A-HA_3_ cds1::ura4+* strains were incubated for 3.5 h at 30°C in rich medium (U), in 12 mM HU (H) or 10 μM CPT (C). Total protein extracts were analysed on a 10% SDS page.

The next decisive observation came when we analysed the S-M checkpoint response to broken DNA replication forks. As long as the structural integrity of a stalled fork is protected by Cds1 kinase, Chk1 activity remains low (Xu et al., 2006). Cds1 (Chk2) kinase shields stalled replication structures from nucleases and recombination enzymes (Kai et al., 2005; Boddy et al., 2003); however, Chk1 is strongly activated when forks break in the absence of Cds1, and cells without Chk1 and Cds1 are completely checkpoint defective (Lindsay et al., 1998). To test whether the S173A mutation impairs this response, we combined the *chk1-S173A-HA_3_* allele with the deletion of *cds1* (*Δcds1*). The double mutant was as HU sensitive as the *Δchk1 Δcds1* strain, strongly implying that the activation loop mutation blocks Chk1 activation when replication forks collapse in the absence of Cds1 (Fig. 5A). This conclusion was confirmed when we released G2-synchronised *chk1-S173A-HA_3_ Δcds1* cells into rich medium with 12 mM HU. Like the checkpoint defective *Δchk1 Δcds1* strain, the *chk1-S173A-HA_3_ Δcds1* mutant entered a fatal mitosis 140 min post-release (Fig. 5B). The majority of cells died while they re-entered the cell cycle, indicated by the cut phenotype where one daughter cell is anuclear or where the new wall cuts through the single nucleus (Fig. 5C). Collectively, these results demonstrate an outright dependency of cells on serine 173 when replication forks break in the absence of Cds1. As in the earlier experiments, Chk1-S173A was fully phosphorylated at S345 in *Δcds1* cells (Fig. 5D). These results imply a defect of Chk1-S173A down-stream of collapsed replication forks in the absence of Cds1. To our knowledge this is the first *chk1* allele with this Cds1-specific defect.

### Chk1-S173A reduces the viability of DNA polymerase epsilon mutant cells

**Fig. 6.**
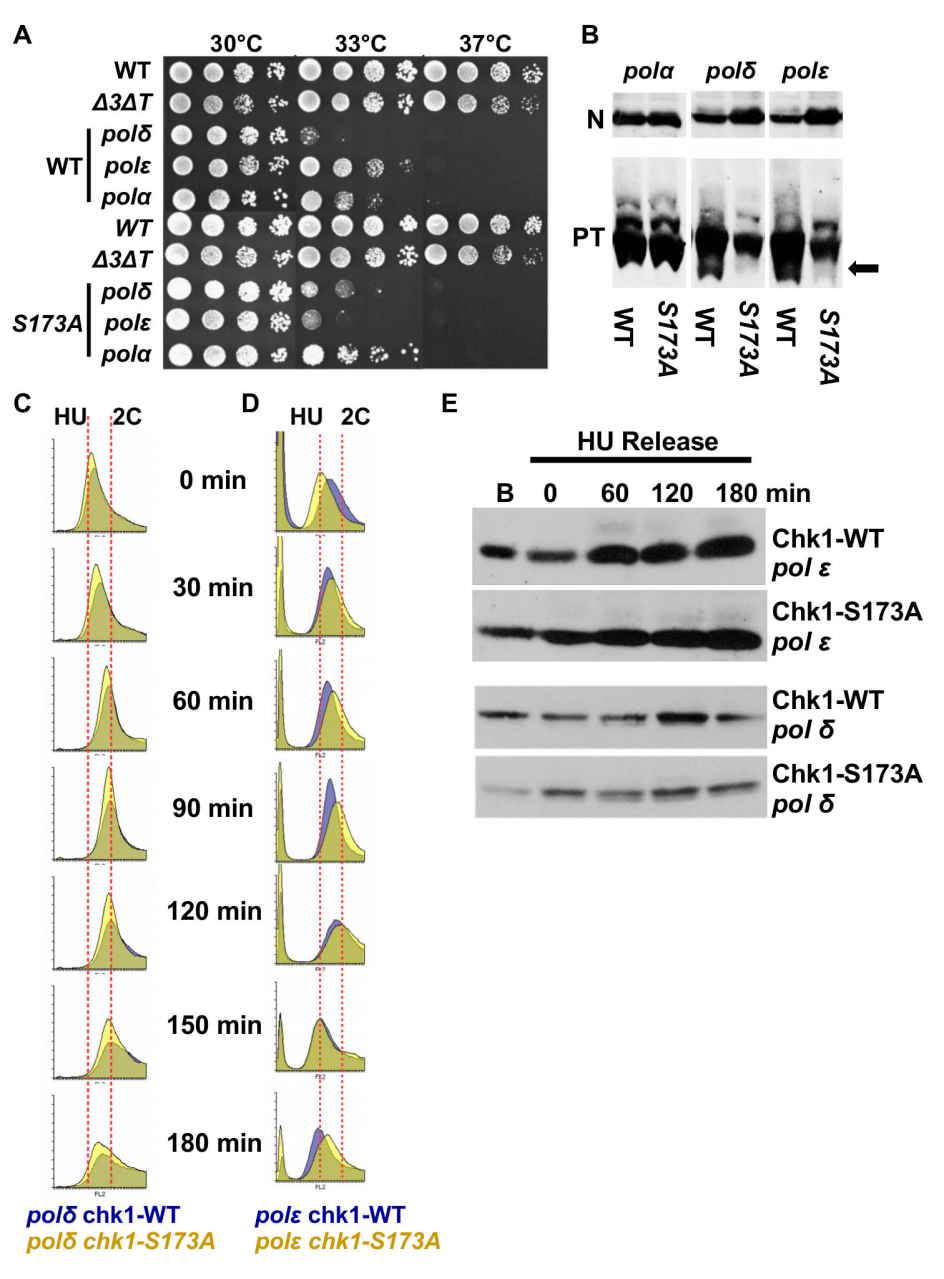
**Chk1-S173A reduces the viability of DNA polymerase epsilon mutant cells.** (A) Drop test with the indicated strains on rich medium plates. The *chk1-HA_3_* (WT) and *chk1-S173A-HA_3_* (S173A) alleles were crossed into *swi7-H4* (pol alpha), *cdc6.23* (pol delta) and *cdc20.M10* (pol epsilon). (B) PT-SDS and normal SDS (N) analysis of the strains used in the drop test. Total protein was extracted after growth in rich medium for 3.5 h at 30°C. The arrow indicates the hypo-phosphorylated Chk1 protein. (C,D) *chk1-HA_3_ cdc6.23*, *chk1-HA_3_ cdc20.M10*, *chk1-S173A-HA_3_ cdc6.23* and *chk1-S173A-HA_3_ cdc20.M10* cells were synchronised in early S phase by incubating cells in rich medium with 15 mM HU for 3.5 h. Flow cytometry histograms were recorded at the indicated times after HU was washed out*.* The dotted lines indicate HU arrested and G2 (2C) cells. (E) Total protein samples were prepared from samples taken from this experiment at the indicated times and analysed on normal SDS page.

Because deletion of *S. pombe chk1* compromises the viability of temperature-sensitive mutants of DNA polymerase delta and epsilon (Francesconi et al., 1995), we combined mutant alleles in the three replicative DNA polymerases alpha (*swi7-H4*), delta (*cdc6.23*) and epsilon (*cdc20.M10*) with either c*hk1-HA_3_* or *chk1-S173A-HA_3_*. While testing cell growth at the semi-restictive temperature of 33°C, we noticed that the S173A mutation specifically reduced the viability of the pol epsilon (*cdc20.M10*) mutant as the *chk1-S173A-HA_3_ cdc20.M10* double mutant grew only very poorly compared to the *chk1-HA_3_ cdc20.M10* strain (Fig. 6A). DNA polymerase epsilon synthesises the leading strand (Pursell et al., 2007), is involved in long-patch BER (Wang et al., 1993), associates with the DNA replication checkpoint protein Mrc1 (Claspin) (Lou et al., 2008), and establishes heterochromatin (Li et al., 2011). The reduced viability at 33°C could suggest two roles of Chk1: either the kinase responds to replication problems associated with the leading strand, or it promotes DNA pol delta that can remove mismatches left behind by pol epsilon (Flood et al., 2015). Phos-tag analysis showed that some hypo-phosphorylated material was absent from Chk1-S173A, but this was the case for both, pol delta and epsilon (Fig. 6B).

We next synchronised the strains in early S phase using the HU protocol (Luche and Forsburg, 2009) and released them back into the cell cycle to follow their progression into G2. While the S173A mutation had no impact in the case of DNA polymerase delta (*chk1-S173A-HA_3_ cdc6.23*) (Fig. 6C), it did advance cell-cycle progression in the DNA polymerase epsilon strain (*chk1-S173A-HA_3_ cdc20.M10*) (Fig. 6D). The mutation in the activation loop allowed cells to acquire a G2 (2 copies, 2C) DNA content 90 min post-release, approximately 30 min earlier than the wild-type Chk1 kinase (*chk1-HA_3_ cdc20.M10*). We did, however, find no evidence of S345 phosphorylation in any mutant strain during this experiment (Fig. 6E). The faster progression of the *chk1-S173A-HA_3_ cdc20.M10* mutant could explain why the pol epsilon strain loses viability at the semi-permissive temperature. The S173A mutation might block the phosphorylation of a down-stream target that is crucial for a reduction in leading strand synthesis when DNA polymerase epsilon is impaired or when pol delta needs to remove mismatched nucleotides.

### The MMS sensitivity of Chk1-S173A is linked with DNA polymerase delta

**Fig. 7.**
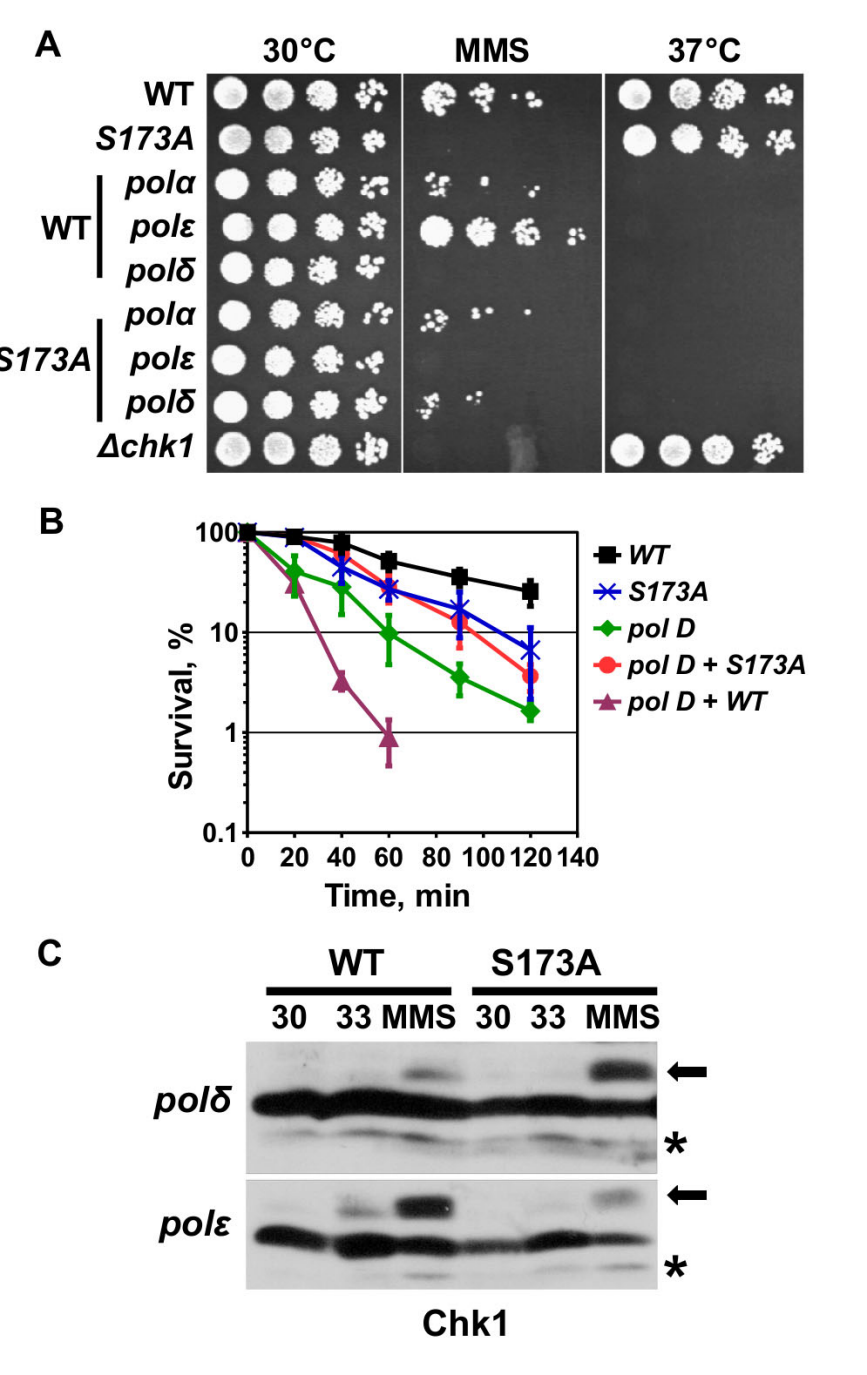
**The MMS sensitivity of Chk1-S173A is linked with DNA polymerase delta.** (A) Drop test with the indicated strains on rich medium plates at 30°C, 30°C with 0.01% MMS, or 37°C. (B) Acute MMS survival (0.025%) at 30°C (mean±s.d. of three repeats). (C) *chk1-HA_3_ cdc6.23*, *chk1-HA_3_ cdc20.M10*, *chk1-S173A-HA_3_ cdc6.23* and *chk1-S173A-HA_3_ cdc20.M10* cells were grown at 30°C, 33°C or 30°C plus 0.01% MMS for 3.5 h in rich medium. Total protein extracts were analysed on normal SDS page.

Given the requirement of pol delta for the removal of alkylated bases by BER (Blank et al., 1994), we tested the genetic relationship between *chk1-S173A-HA_3_* and *cdc6.23*. Intriguingly, the mutation in the catalytic subunit of pol delta affected survival on MMS plates differentially depending on whether the *chk1-HA_3_* wild-type or *chk1-S173A-HA_3_* mutant allele was present. While *cdc6.23* cells containing the wild-type kinase were MMS sensitive, *cdc6.23* cells with the mutant kinase displayed some degree of resistance (Fig. 7A). We followed this observation up by conducting an acute survival test at 0.025% MMS and noticed that the *chk1-HA_3_ cdc6.23* double mutant was significantly more MMS sensitive than the pol delta (*cdc6.23*) single mutant that contains the untagged *chk1* gene (Fig. 7B). This implies that the tagged *chk1-HA_3_* allele, which has been used in many studies (Walworth and Bernards, 1996), differs from the untagged gene in a *cdc6.23* mutant background. Intriguingly, the mutation in the activation loop suppressed this hyper-sensitivity to a level observed for the *chk1-S173A-HA_3_* single allele (Fig. 7B). Collectively, these data show that the MMS sensitivity of the *chk1-S173A* mutation is epistatic with the *cdc6.23* mutation in the catalytic subunit of pol delta at 30°C and that the mutation also suppresses the damaging activity of the tagged wild-type Chk1 kinase. The nature of this activity is as yet unknown. We suspect however that the C-terminal tag interferes with the repair function of pol delta in BER (Blank et al., 1994). To test whether the polymerase mutations interfere with S345 phosphorylation of Chk1 and Chk1-S173A, the corresponding strains were treated with 0.01% MMS at 30°C and also exposed to the semi-permissive temperature of 33°C without MMS. While both Chk1 proteins were phosphorylated at S345 in the presence of MMS, the phosphorylation of the wild-type kinase was lower in the pol delta mutant coinciding with its high MMS sensitivity (Fig. 7C). Chk1 was only weakly S345 modified at 33°C in both polymerase mutants indicating that no or very little endogenous DNA damage occurs under these conditions.

## DISCUSSION

**Fig. 8.**
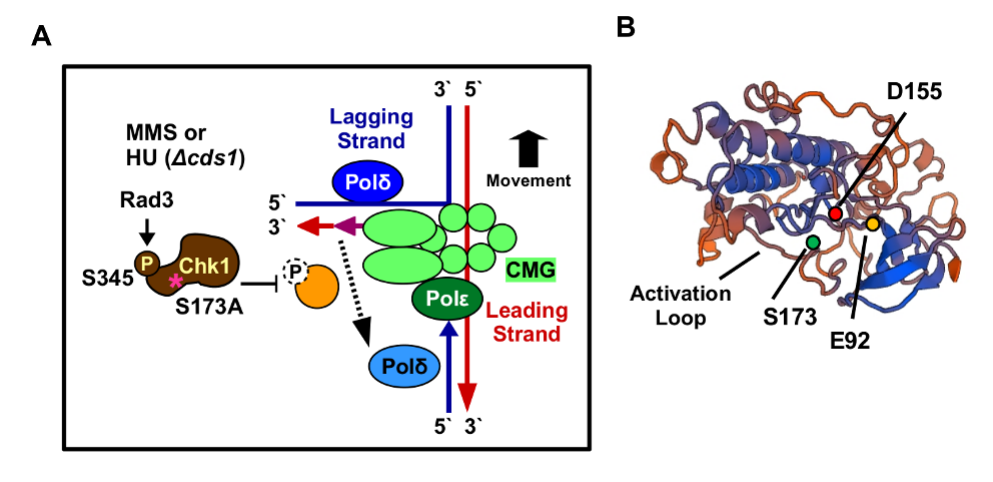
**Model of how Chk1-S173 affects DNA polymerase delta** (A) Chk1-S173A may be defective in the phosphorylation of a protein that allows DNA pol delta, which acts in front of the moving replication fork on the lagging strand, to remove mismatches that remain in the leading strand in the presence of MMS. Since pol delta also synthesis both strands during the recombinogenic repair of collapse forks, Chk1-S173A might also impair this function. CMG=Cdc45+ Mcm2–7+GINS replication complex. (B) Model of the kinase domain of *S. pombe* Chk1. The underlying crystal structure is 4czt (34.5% identity).

The only separation-of-function conditions known so far are the phosphorylation of S317 of human Chk1, which is only required for the DNA damage response but not for its essential functions (Wilsker et al., 2008), and the mutations E92D and I484T in *S. pombe* Chk1 which affect the S-M checkpoint but only at 37°C (Francesconi et al., 1997). We report here a new separation-of-function mutation, S173A, in the kinase domain of *S. pombe* Chk1, that abolishes the G1-M arrest in a *cdc10.V50* strain background and the S-M checkpoint in HU-treated *Δcds1* cells without affecting the G2-M arrest in the presence of exogenous DNA damaging agents. When *chk1-S173HA_3_* cells arrest at start, during the G1-S transition due to the *cdc10.V50* mutation, they cannot prevent mitosis (Fig. 4C,D). A similar problem arises when DNA replication forks break in HU medium in the absence of Cds1 (Fig. 5B,C). Since *cdc10.V50* cells arrest with unreplicated chromosomes at start (Luche and Forsburg, 2009), both Chk1 requirements must reflect distinct G1-M and S-M checkpoint activities of Chk1. What is, however, intriguing is that the *chk1-S173A* mutant is not CPT sensitive (Fig. 2E), although camptothecin also breaks DNA replication forks (Pommier et al., 2010). This implies that the S173A mutation is only critical when Cds1 is absent. Since Cds1 protects damaged forks from nucleases and recombinases (Kai et al., 2005; Boddy et al., 2003), it is possible that the mutation affects a DNA repair factor that is redundant as long as Cds1 is active. The DNA damage signal must reach Chk1-S173A as the mutant kinase is phosphorylated at S345 in the presence of CPT (Fig. 1D), 4-NQO (Fig. 1D), MMS (Fig. 2H) and HU (Fig. 1D). It is therefore unlikely that the S173A mutation interferes with Rad3 activation at damaged chromosomes involving Crb2 (53BP1), Rad4 (TopBP1) and the 9-1-1 ring (Furuya et al., 2004). Since the corresponding lysine-166 in human Chk1 is involved in substrate specificity (Chen et al., 2000), it is more likely that the S173A mutation blocks the phosphorylation of a down-stream target that is required to restrain mitosis in *cdc10.V50* mutant cells and when forks break in *cds1* deletion cells (Fig. 8A). This target appears to be distinct from Wee1 and Cdc25 because the *chk1-S173A* strain is able to block mitosis when *cds1+* cells are treated with HU or the UV mimetic 4-NQO (Fig. 2B,C). A clear difference exists, however, when DNA is alkylated at high MMS concentrations (0.05%) as *chk1-S173A* cells have a partial G2-M checkpoint defect (Fig. 2D and Fig. 3C) and are sensitive (Fig. 2G and Fig. 3D). Since MMS introduces single-strand DNA damage, the dose-dependency of the checkpoint defect and the sensitivity implies a role of S173 only when these lesions reach a high threshold. Such a threshold may be the conversion of single-strand lesions in close proximity into double-strand breaks or a more pronounced arrest of replication forks when both DNA strands are alkylated.

The genetic link between DNA polymerase delta and Chk1-S173A may hint at the unknown target that cannot be activated in a *chk1-S173A* strain. The observation that the kinase domain mutation reduces the viability of the pol epsilon (*cdc20.M10*) mutant at 33°C (Fig. 6A) could be explained by the faster progression through S phase (Fig. 6D). This faster progression may however be linked with DNA polymerase delta given that pol epsilon needs pol delta to repair any remaining mismatches in the leading strand which are not removed by its own 3′-exonuclease activity (Flood et al., 2015). Pol delta is also able to replicate the leading and the lagging strand once a fork has collapsed (Miyabe et al., 2015). The requirement of S173 for viability of the pol epsilon (*cdc20.M10*) mutant could therefore mean that Chk1 is involved in the repair activities of pol delta either when mismatched bases remain in the leading strand after MMS treatment or when the leading strand is elongated by pol delta during the homologous recombination dependent re-start of collapsed replication forks in HU-treated *Δcds1* cells (Fig. 8A). This conclusion is strengthened by the epistatic relationship between *chk1-S173A* and *cdc6.23*, the catalytic subunit of pol delta (Fig. 7B).

A second interesting characteristic of the S173A mutation is the separation of the S345 phosphorylation from endogenous DNA lesions that normally trigger Chk1 modification by Rad3 (Fig. 1G). Although S345 is normally phosphorylated in *chk1-S173A* cells after exposure to exogenous DNA damaging agents (Fig. 1D), it is only very poorly modified during unperturbed growth (Fig. 1G; Fig. S2C,D). Since DNA replication stress is the most likely source of endogenous damage in growing cells, the S173A mutation appears to reduce this risk, but how? The best explanation provides the requirement of mammalian Chk1 for the regulation of the replication speed indirectly through its control of origin firing (Petermann et al., 2010). An increase in active origins reduces replication speed and if fast replication in wild-type cells is the source of the endogenous Chk1-S345 phosphorylation, the S173A mutation may suppress this modification by being defective in the suppression of origins. In other words, a higher number of active origins in the S173A strain may alleviate replication stress by slowing down fork progression.

In summary, S173A is a rare separation-of-function mutation of Chk1 that may help to dissect its role in S phase where it might link post-replication repair by DNA polymerase delta with a block over mitosis. The identification of its proposed target will, however, require more work. It is intriguing that one of the other known separation-of-function mutations, E92D (Francesconi et al., 1997), sits at the beginning of a loop opposite the activation loop where S173A is (Fig. 8B).

## MATERIALS AND METHODS

### Yeast strains

The genotype of the strains used in this study is *ade6-M210 leu1–32 ura4-D18*. The *rad3* gene was deleted with the *ade6+* gene and the *cds1* gene was deleted with *ura4+*. The *chk1* gene was deleted with *kanMX4* antibiotic resistance gene, *chk1-S345A-HA_3_* *(h- ade6-M210 chk1::loxP-chk1-S345A-HA3-loxM leu1-32 ura4-D18); chk1-S173A-HA_3_ (h- ade6-M210 chk1::loxP-chk1-S173A-HA3-loxM leu1-32 ura4-D18)* (Fig. S1)*.* See figure legends for further details.

### Base strain construction and integration of the Chk1 point mutations

The base strain was constructed as described in Watson et al. (2008). The *loxP* and *loxM* Cre-recombinase recognition sequences were integrated 84nt upstream of the start codon and 84nt downstream of the stop codon (Fig. S1A) using the primers *Base-1* and *Base-2* (Fig. S1C). The point mutations S173A and S345A were introduced using fusion PCR as reported in Janes et al. (2012). Genomic DNA from the c*hk1-HA_3_* strain (Walworth and Bernards, 1996) was used as the PCR template to introduce the C-terminal HA affinity tag. The two overlapping *chk1* gene segments were amplified using the primers *Base-3* and the mutation reverse primer, and the primer *Base-4* and the mutation forward primer (Fig. S1C). The full-length fusion fragments were cloned into the *lox*-Cre integration plasmid using the restriction enzymes SphI and SacI. Integration of the mutated *chk1-HA_3_* genes resulted in the loss of 4nt upstream of the start codon and of 17nt downstream of the stop codon (Fig. S1B).

### Cell synchronisation

Cells were synchronised as described in Luche and Forsburg (2009). HU was used at a final concentration of 15 mM for 3.5 h at 30°C in rich medium. Lactose gradients were centrifuged for 8 min at 800 rpm. The *nda3.KM311* mitotic arrest was performed in rich medium as reported in Nakazawa et al. (2011). One volume of pre-warmed medium (40°C) was added to the 20°C medium to quickly raise the temperature to 30°C at the up-shift to re-start the cell cycle.

### Flow cytometry

The DNA content was measured using a CUBE 8 (Sysmex) instrument as described in Luche and Forsburg (2009). The histograms were produced using the free Flowing Software (http://flowingsoftware.btk.fi/).

### Phos-tag SDS page

Phostag gels (6%) were prepared and run as reported in Caspari and Hilditch (2015).

### Survival assays

The drop tests and acute survivals assays are described in Kai et al. (2006).

### Antibodies

Anti-HA antibody (BioScource, Covance MMS-101P-200) was used.

## Supplementary Material

Supplementary information
