# Supplementary material for: The kinase domain residue serine 173 of Schizosaccharomyces pombe Chk1 kinase is critical for the response to DNA replication stress
Source: Biol Open. 2017 Nov 1;6(12):1840–50. doi: 10.1242/bio.029272 (PMC5769658; doi:10.1242/bio.029272)
Supplement: Supplementary information [file biolopen-6-029272-s1.pdf]

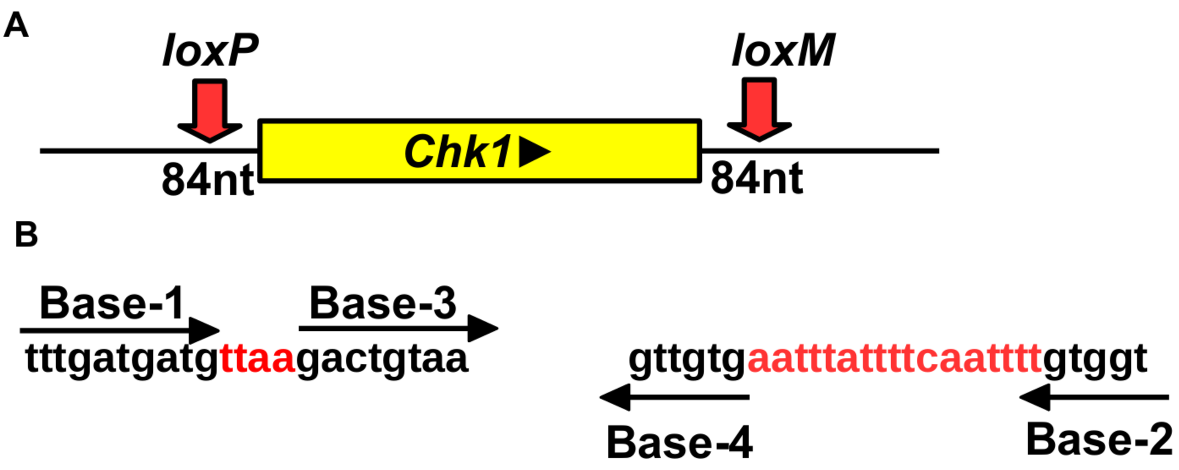

C

Chk1-Base-1
GTCCAATTTTATATCATTATATAGATTTAAAACTCATTTAAAAATTA CCTTAATTTAATCTTTTCCAGTCTTTGATGATG cgg atc ccc ggg tta att aa

Chk1-Base-2
CTTCAGCATTCGCAACCATTGATATTTGCTTGACAAAGCGTTAAT AATCCTATTCCCTAGGATATCATTTGTACTACCAC gaa ttc gag ctc gtt taa ac

Chk1-Base-3 (SphI)
atc cta GCATGC atc cta GCAGC GACTGTAAATAATACACATGCC

Chk1-Base-4 (SacI)
gctatc GAGCTC gctatc GAGCTC CACAACGAAGTCAAACAACAGG

Chk1-S173A-F gtaaaagtaggcttttgaatGCtccagtgggtagtccacc

Chk1-S173A-R
GGTGGACTACCCACTGGAGCATTCAAAAGCCTACTTTTAC

Chk1-S345A-F gatcgtgtagaagtttatggcgccttaGcccagccagtacaac

Chk1-S345A-R
GTTGTACTGGCTGGGCTAAGGCGCCATAAACTTCTACACGATC

Supplementary Figure 1. Construction on the *chk1* base strain. (A) Location of the *loxP* and *loxM* sequences upstream and downstream of the *chk1* (SPCC1259.13) gene on chromosome III. (B) Location of the indicated primers. The nucleotides shown in red are deleted in the tagged *chk1* alleles. (C) Primer sequences.

Biology Open • Supplementary information

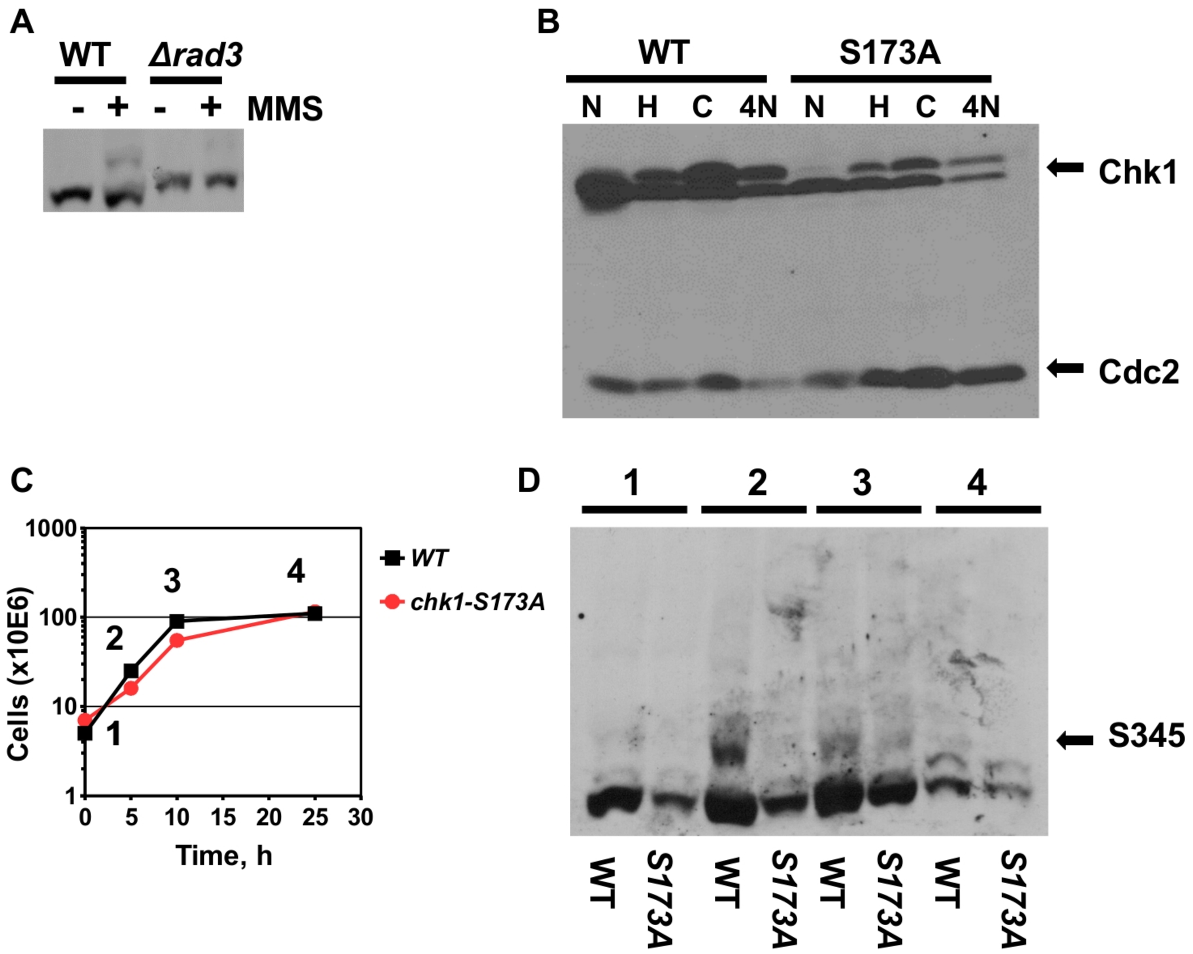

Supplementary Figure 2. (A) *chk1-HA*$_3$ and *chk1-HA*$_3$ *rad3::ade6*$^+$ strains were treated with 0.05% MMS for 1h at 30°C or left untreated. Total protein extracts were analysed on SDS page. (B) Full image of the western blot shown in Fig. 1D. The membrane was first probed with an anti-HA and then with an anti-Cdc2 antibody. (Chk1 runs at 58kDa and Cdc2 at 38kDa). (C, D) Repeat of the experiment shown in Fig. 1F, G.
